# Healthcare utilization of patients accessing an African national treatment program

**DOI:** 10.1186/1472-6963-7-80

**Published:** 2007-06-07

**Authors:** Guy Harling, Catherine Orrell, Robin Wood

**Affiliations:** 1 The Desmond Tutu HIV Centre, Institute for Infectious Disease and Molecular Medicine, Faculty of Health Sciences, University of Cape Town, South Africa

## Abstract

**Background:**

The roll-out of antiretroviral therapy (ART) in Africa will have significant resource implications arising from its impact on demand for healthcare services. Existing studies of healthcare utilization on HAART have been conducted in the developed world, where HAART is commenced when HIV illness is less advanced.

**Methods:**

This paper describes healthcare utilization from program entry by treatment-naïve patients in a peri-urban settlement in South Africa. Treatment criteria included a CD4 cell count <200 cells/μl or an AIDS-defining illness. Data on health service utilization were collected retrospectively from the primary-care clinic and secondary and tertiary referral hospitals. Hospital visits were reviewed to determine the clinical reason for each visit.

**Results:**

212 patients were followed for a median of 490 days. Outpatient visits per 100 patient years of observation (PYO), excluding scheduled primary-care follow-up, fell from 596 immediately prior to ART to 334 in the first 48 weeks on therapy and 245 thereafter. Total inpatient time fell from 2,549 days per 100 PYO pre-ART to 476 in the first 48 weeks on therapy and 73 thereafter. This fall in healthcare utilization occurred at every level of care. The greatest causes of utilization were tuberculosis, cryptococcal meningitis, HIV-related neoplasms and adverse reactions to stavudine. After 48 weeks on ART demand reverted to primarily non-HIV-related causes.

**Conclusion:**

Utilization of both inpatient and outpatient hospital services fell significantly after commencement of ART for South African patients in the public sector, with inpatient demand falling fastest. Earlier initiation might reduce early on-ART utilization rates.

## Background

The past two years have seen the rapid expansion of access to highly-active antiretroviral therapy (ART) worldwide, with the aim of treating all 6.5 million people who are estimated to need treatment [1]. South Africa has also seen a rapid expansion of those taking antiretrovirals through the public sector since the public sector ART program was begun in 2004 [2]. It has been argued that the most pressing constraint on the progress of this expansion of access will be a lack of healthcare personnel and other resources [3,4], but there remains little evidence on the impact of ART provision on healthcare utilization in settings where HIV is a significant burden on the healthcare system [5,6].

A large proportion of those accessing healthcare in the public sector in Africa are HIV positive. In 1992, 39% of patients at the main tertiary referral hospital in Nairobi were HIV positive, and by 1997 this had risen to 70% of medical admissions [7,8]. Studies in South Africa found that the number and proportion of patients being admitted for diagnoses often linked to advanced HIV-related disease increased considerably between 1991 and 2002 [9], and that inpatient seroprevalence rates were over 50% in Durban by 1998 [10]. Information on the use of services both prior to and after the commencement of ART, and by patients at different stages of illness, is needed to accurately predict future resource needs.

This increased burden of acute illness due to HIV has placed significant strain on healthcare systems in the region. The experience of North America was that the advent of ART led to a fall in demand for inpatient care [11-14]. Its effect on demand for outpatient care is unclear, with some studies finding an increase in demand [11,12], while others found a decrease [13,14]. One study noted that the decrease in inpatient demand was particularly steep for HIV-related inpatient stays [14]. These changes are likely to have been due to reduced need to treat opportunistic infections (OIs) [15]. A recent hospital-based study of the cost-effectiveness of ART in South Africa found a partial offsetting of additional medicine costs for ART by reduced hospital inpatient stays and outpatient visits [16].

There has to date, however, been no research on healthcare utilization at the commencement of ART in Africa. In more developed nations commencement of antiretrovirals is not associated with severe illness or with significant changes in overall healthcare utilization. Data from such settings are unlikely to be predictive of utilization rates in Africa, however, since patients in richer nations commence ART far earlier in the HIV disease process, avoiding the potential for high levels of mortality and morbidity associated with commencing ART at CD4 counts under 100 cells/μl [17,18].

The aim of this study is to assess patterns of healthcare utilization seen as severely immune-suppressed persons begin ART at a primary-care clinic in Africa. This information will contribute to the planning of healthcare provision as access to antiretrovirals is expanded.

## Methods

### Data collection

The Hannan Crusaid Treatment Centre (HCTC) is a dedicated, public-sector antiretroviral clinic in a peri-urban settlement near Cape Town, South Africa. The site began recruitment in September 2002, and its work has been previously described [19]. For those enrolled at the centre, the HCTC acts as their primary health care facility, dispensing ART and providing treatment for HIV-related OIs and other illnesses.

Patients are referred by local primary-care providers based on CD4 lymphocyte count or clinical symptoms of AIDS. They are screened at the HCTC and seen a second time 14 days later, prior to commencing ART. Patients then attend scheduled visits at 0, 4, 8 and 16 weeks after commencement, and every 16 weeks thereafter. Patients may make unlimited unscheduled visits to the HCTC as required.

Any referrals to higher levels of the public health system are recorded, and patients are referred back to the HCTC after such care has been received. All public-sector care is free at the point of service, although non-primary utilization requires a referral in order to qualify for this.

The study population was eligible patients who enrolled in the program prior to January 2004. In line with national guidelines eligibility criteria were a CD4 count of less than 200 cells/μl or an AIDS-defining illness [20]. In addition, the HCTC required a local residential address, attendance of three treatment education sessions and disclosure to at least one friend or relative. All subjects were followed-up for a minimum of 48 weeks or to censor; those who commenced antiretroviral therapy prior to October 2003 were additionally followed until their final scheduled four-monthly visit prior to January 2005. Patients were right censored for death, loss to follow-up or transfer to another ART site.

The primary regimen was non-nucleoside reverse transcriptase (NNRTI) based and consisted of stavudine, lamivudine and efavirenz (or nevirapine if pregnancy was likely). In the case of virological failure or of adverse reaction a secondary, protease inhibitor (PI) based regimen of zidovudine, didanosine and lopinavir/ritonavir was provided.

Data on patient-specific service utilization at the HCTC were collected prospectively from patient files kept at the centre. Data on service utilization at hospitals in Western Cape were collected retrospectively from paper-based medical records at the relevant hospitals. Where such files were not available, the lengths of stay and laboratory tests performed were identified from less detailed, computerized records. Ethical approval for this study was gained from the University of Cape Town Research Ethics Committee and all patients gave informed consent.

### Data analysis

Hospital visits were first categorized, according to the South African Department of Health's classifications of hospitals, into tertiary, secondary and specialist institutions. Hospital visits were second categorized according to the primary reason for seeking care, based on information available from patient's files. Visits were separated into those due to: (i) HIV-related illness, by OI; (ii) adverse reactions to antiretrovirals, by probable causative drug; (iii) health problems unrelated to HIV, by primary site of illness; and (iv) investigations of symptoms.

Visits were further categorized into five periods by timing relative to the commencement of ART: pre-ART; 0 to 16 weeks on ART, 17 to 32 weeks on ART, 33 to 48 weeks on ART and all time from 49 weeks to end of follow-up. Maximum patient follow-up was 112 weeks on ART. Visit rates were calculated as numbers of outpatient visits and inpatient days per 100 patient years of observation (PYO).

Continuous data were described using medians and interquartile ranges (IQRs); categorical data using counts and percentages. Differences in continuous data were evaluated using the Rank-Sum test; trends in rates were evaluated using the Cochran-Armitage test. All tests were two-sided at α = 0.05.

## Results

### Study population and overall utilization

253 persons were screened at the clinic in 2002 and 2003. Prior to ART commencement 41 persons were either lost to follow-up or excluded as not meeting the eligibility requirements. The study population thus consisted of 212 patients, whose baseline demographic, clinical and virological characteristics are described in Table 1. Twelve patients died prior to commencing ART, 200 patients started ART, 172 were followed-up to 48 weeks and 132 to at least 64 weeks. The median period of follow-up pre-ART was 30 days (IQR 28–50) and on ART was 448 days (IQR 336–672).

**Table 1:** Demographic, clinical and virological characteristics on admission to program

Age, median (IQR) in years	33	(29–38)
		
Sex		
Female	153	(72.5)
Male	58	(27.5)
		
WHO clinical stage		
1 or 2	22	(10.4)
3 (Symptomatic)	97	(45.8)
4 (AIDS)	93	(43.8)
		
CD4 count (cells/μl)		
≤ 50	66	(31.3)
51 – 100	58	(27.5)
101 – 200	71	(33.6)
> 200	16	(7.6)
		
HIV RNA (copies/ml)		
≤ 1000	3	(1.5)
1001 – 10 000	13	(6.4)
10 001 – 100 000	91	(45.1)
> 100 000	95	(47.3)

Figures are numbers (percent) unless otherwise stated. N = 212.

During the 279 patient-years observed, 2573 outpatient visits and 1494 inpatient days, consisting of 109 separate admissions, were recorded (Table 2). One tertiary, four secondary and one dedicated tuberculosis hospital were attended. The overall rate of visits, excluding scheduled primary-care ART monitoring visits, fell from 596 per 100 PYO in the pre-ART period to 334 per 100 PYO during the first 48 weeks on ART and further to 245 per 100 PYO thereafter. The total rate of inpatient days also fell from 2549 days per 100 PYO prior to starting ART to 476 days per 100 PYO in the first 48 weeks and 73 days per 100 PYO subsequently.

**Table 2:** Distribution of visits within the cohort: rates per 100 patient-years of observation (absolute numbers in parentheses)

					**Outpatient Visits**				**Inpatient Stays**				
							
**Period on ART**	**Initial no. of Patients^a^**	**Patient Years**	**Deaths**	**Losses to Program^b^**	**HCTC Unscheduled**	**HCTC Scheduled**	**Tertiary**	**Total**	**Tertiary**	**Secondary**	**Tuberculosis**	**Total**	**Patients with no inpatient stay (%)**
Pre-treatment	211	25	48 (12)		382 (96)	1662 (418)	115 (29)	2258	243 (61)	225 (64)	2051 (516)	2549	89
0–16 weeks	200	58	26 (15)	7 (4)	312 (181)	996 (557)	86 (50)	1427	294 (170)	478 (277)	130 (75)	902	83
17–32 weeks	181	55	4 (2)	7 (4)	174 (95)	331 (181)	59 (32)	593	187 (102)	31 (17)		218	94
33–48 weeks	175	53	6 (3)		211 (112)	352 (187)	55 (29)	654	75 (40)	186 (99)	15 (8)	276	91

49–64 weeks	132	89	2 (2)	1 (1)	173 (153)	328 (290)	46 (41)	573	38 (34)	35 (31)		73	94
65–80 weeks	89												
81–96 weeks	54												
97–112 weeks	15												

Total	212	279	12 (34)	3 (9)	228 (637)	592 (1653)	63 (181)	920	148 (407)	175 (488)	214 (599)	537	62

Figures presented in the text may not be precisely reproducible from the data provided due to rounding. ^a ^One patient commenced ART at her first visit and therefore does not appear in the Pre-treatment period. ^b ^Losses to program consisted of eight losses to follow-up and one transfer out occurring at 0–16 weeks.

In the pre-ART period 65 patients (31%) made an unscheduled primary-care visit, 19 (9%) had attended a secondary care clinic and 14 (7%) had appointments at the tertiary level. Five (2%) of patients had a tertiary inpatient episode in the period prior to commencing ART and 18 (9%) had a secondary inpatient visit. During the 48 weeks on ART 128 (61%) patients made unscheduled visits to the HCTC, while only 26 (13%) attended secondary clinics and 32 (16%) attended tertiary clinics. Forty-one (21%) patients had a secondary inpatient episode during their first 48 weeks on ART and 14 (7%) had a tertiary inpatient episode. A summary of causes of hospital utilization is provided in Table 3.

**Table 3:** Summary of major causes of hospital care utilization prior to and on ART

	**Outpatient visits**			**Inpatient days**		
		
Hospital type	**Secondary**	**Tertiary**	**Total (%)**	**Secondary**	**Tertiary**	**Total (%)**
Tuberculosis & Bronchiectasis	6			703 ^1^		
Other bacterial infections	4	7		40	12	
Cryptococcal Meningitis	14			90	42	
Other fungal infections	7	3		28		
Kaposi's sarcoma		33				
Other HIV-related neoplasms	1	8			173	
Protozoan infections	3	3		19	23	
Warts		10			11	
CMV Retinitis		15			4	
Other Viral infections	1	15		1	5	
Other HIV-related events	2	10			17	
All HIV-related events			142 (50)			1168 (78)

						
Efavirenz reaction				5	13	
Nevirapine reaction				1	21	
Stavudine reaction	11			56	19	
All ART drug reactions			11 (4)			115 (8)

						
Investigations ^2^	14		14 (5)			-

						
Antenatal care	4	8		7	21	
Gynecological		7			9	
Hepatic	6	12		16	6	
Ophthalmic		19			5	
Cardio-vascular	5			46		
Other	24	31		75	26	
All non-HIV-related events			116 (41)			211 (14)
		
Total	102	181		1087	407	

^1 ^Includes admissions to specialist tuberculosis hospital. ^2 ^Cause remained uncertain after investigation.

### Outpatient care

The vast majority (89%) of all outpatient visits were made to the primary healthcare provider, the HCTC. Of these, almost three-quarters (72%) were scheduled visits, falling most heavily in the periods pre- and immediately post-initiation of ART. Once commenced on ART the rate of both scheduled and unscheduled visits fell until 17 to 32 weeks on ART, after which they stabilized. The rate of both types of visit had a downward trend (χ^2 ^= 517 & χ^2 ^= 41.6, p < 0.001).

**Figure 1:**
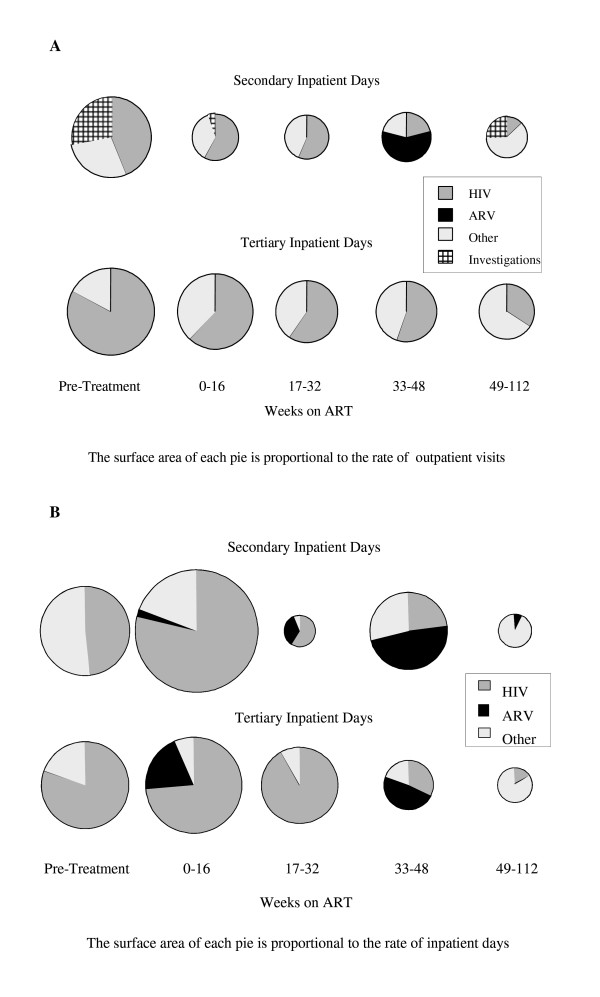
Breakdown at each hospital level by cause of demand of (a) outpatient visits and (b) inpatient days.

Secondary outpatient visit rates were highest prior to commencement of ART, falling rapidly once ART began; there was a significant downward trend in these rates (χ^2 ^= 10.0, p < 0.002). Once ART had commenced, the majority of visits were for HIV-related illnesses up to 32 weeks (Figure 1a). From 32 to 48 weeks on ART over half of visits (58%) were for ART-related illnesses relating to stavudine, either lactic acidosis or peripheral neuropathy. After 48 weeks on ART most visits (61%) were for non-HIV-related matters.

The rate of tertiary outpatient visits was highest prior to ART, declining to a plateau after 32 weeks on ART. The majority of these visits from screening to 48 weeks on ART were for HIV-related illnesses; after 48 weeks this proportion declined to a third of the total. The single largest cause of HIV-related visits was treatment of Kaposi's sarcoma and other cancers (39%) while the largest cause of non-HIV-related visits was ophthalmic complaints (25%). Like all other inpatient and outpatient categories, tertiary visit rates declined over time (χ^2 ^= 15.6, p < 0.001).

### Inpatient care

There was a downward trend in the overall rate of inpatient days (χ^2 ^= 1463, p < 0.001). A large proportion of all secondary care inpatient days were due to HIV-related illness up to 32 weeks on ART (Figure 1b). Key causes were tuberculosis and bronchietasis (41%) and Cryptococcal meningitis (31%). This burden of HIV-related care overlapped with stavudine-related illness, which accounted for 46% of all secondary inpatient days between 17 and 48 weeks on ART. After 48 weeks on ART the large majority (90%) of care was due to non-HIV-related illness.

The majority of tertiary inpatient time over the first three periods of observation was due to HIV-related illness. Over half (56%) of all these days were due to one patient being treated for Burkitt's lymphoma. One fifth of all inpatient time in first 16 weeks on ART was due to two patients, one suffering an adverse reaction to efavirenz, the other to nevirapine, although this latter stay was extended by a nosocomial infection. Between 32 and 48 weeks almost half (48%) of all tertiary inpatient care was due one patient suffering from an adverse reaction to stavudine. Once more, after 48 weeks on ART most inpatient care (84%) was for non-HIV-related issues.

There were six admissions to the specialist tuberculosis hospital prior to ART commencement, with a mean stay of 86 days; a further two admissions arose on ART, with a mean stay of 42 days. These admissions accounted for 40% of all inpatient days recorded.

An analysis excluding the eight patients attending the dedicated tuberculosis hospital and the four patients individually identified as the heaviest users of services above still found a downward trend in service utilization over time. The rate of inpatient days fell to 496 per 100 PYO in the pre-ART period and to 506 per 100 PYO in the first 16 weeks on ART. Thereafter rates were similar to the main results. A sub-analysis looking at those 12 patients who died prior to commencing ART found their use of inpatient services to be far greater than other patients (16,128 versus 1954 days per 100 PYO in the pre-ART period). Their use of outpatient services was, however, almost identical to the cohort as a whole in the period (569 versus 597 visits per 100 PYO).

## Discussion

The patients in this community-based ART program had a high demand for healthcare services immediately prior to and post initiation of ART. Demand for healthcare fell as time after ART commencement increased, although tertiary and secondary inpatient days (excluding dedicated tuberculosis care) rose briefly in the first four months on treatment. The fall in inpatient care mirrors that seen elsewhere, but the fall in outpatient care is in contrast to findings both in North America and in a hospital-based ART program in South Africa [21,22]. Removing the frequent scheduled primary care visits required prior to commencement does not reverse this latter effect.

Although the clinical characteristics of these patients are not identical to those reported from North America, a comparison may be instructive. The rates of utilization seen in this population prior to ART initiation, both for inpatient days (2258 per 100 PYO) and outpatient visits (2549 per 100 PYO), were far higher than those reported elsewhere [12-14]. On ART, outpatient utilization (920 visits per 100 PYO) was comparable to North American rates, although inpatient demand (537 days per 100 PYO) was almost double that seen elsewhere. Given the high levels of viral suppression in this population [23], this higher rate of utilization is unlikely to reflect poor viral suppression. It may, however, reflect either the more advanced illness in this population at ART commencement compared to North American ones, or higher levels of exposure to pathogens in the South African environment.

The utilization of hospital services in this sample was unbalanced: the proportion of patients using inpatient services, shown in Table 3, was low in all periods, and almost two-thirds of patients had no inpatient stay. A small number of patients with HIV-related illnesses which arose prior to 32 weeks on ART were responsible for the majority of the inpatient utilization. Six individuals treated for tuberculosis as inpatients accounted for 81% of all inpatient days pre-ART; two patients with lymphomas consumed 43% of all tertiary inpatient care recorded. Other sizeable causes of hospital care use included Cryptococcal meningitis, Kaposi's sarcoma and cytomegalovirus-related retinitis. Tuberculosis and bronchiectasis accounted for 47% of all inpatient days and other AIDS-defining illnesses a further 28%.

Given this unbalanced distribution of utilization, an intervention that could pinpoint and intensively monitor patients likely to require hospital services might reduce utilization through early treatment of serious conditions. Although overall utilization of hospital services was higher in almost all periods for those patients with low baseline CD4 counts (Table 4), there was no significant difference in baseline CD4 count, viral load or WHO stage between those who did and did not access hospital services in this population (not shown).

**Table 4:** Comparison of overall hospital inpatient and outpatient visit rates stratified by CD4 count

	CD4 count ≤ 50 cells/μl			CD4 count > 50 cells/μl		
		
Period on ART	Initial no. of Patients	Inpatient Days/100 PYO	Outpatient Visits/100 PYO	Initial no. of Patients	Inpatient Days/100 PYO	Outpatient Visits/100 PYO
Pre-treatment	67	3838	253	144	1945	199
0–16 weeks	61	1315	182	139	691	93
17–32 weeks	51	53	112	130	281	79
33–48 weeks	47	645	132	128	139	75
49–112 weeks	38	214	109	94	16	57

CD4 count refers to that recorded at admission to program. PYO: Patient years of observation.

A more proactive intervention would be to initiate ART prior to the onset of clinical symptoms, and thus decrease utilization by reducing the incidence of OIs. In South Africa, the incidence of tuberculosis among HIV infected individuals rises above that seen in the rest of the population once CD4 counts fall below 350 cells/μl [24], and ART reduces this incidence by up to 90% [25,26]. This suggests that commencing ART earlier than the current guideline of less than 200 cells/μl could significantly reduce the proportion of ART-commencing patients requiring acute treatment. The argument is further supported by the observation above that late commencement of ART appears to be associated with increased demand for inpatient care.

Between 33 and 48 weeks on ART, HIV-related demand fell, but there was a rise in service utilization for ART-related illness. In particular, seven patients suffered from either lactic acidosis or peripheral neuropathy, attributed to stavudine, which was severe enough to cause them to visit a hospital, six of them as inpatients. The use of stavudine as a first-line therapy in South Africa is the product of cost constraints. The evidence in this setting suggests that the resource requirements associated with stavudine-related illness may offset some or all of the cost benefits of using stavudine. After twelve months on ART, both HIV-related and ART-related illnesses diminished.

There are a number of limitations inherent to the design of this study. First, the sample size of 212 persons is small, particularly given the skewed nature of the data. The possibility that the patterns seen are driven by a few outlying values cannot be entirely dismissed. Furthermore, the two-year follow-up of this study means that little can be said about the possible displacement of healthcare demand to future periods. Finally, the short pre-ART observation period may have made these results sensitive to patients' recent prior medical history. This latter effect may have been to overestimate demand, if referral to the HCTC was because of recent OI diagnosis, or to underestimate it, if referral followed a recent hospital discharge. These limitations are at least partly the result of the retrospective nature of the study. The inclusion of prospective hospital utilization studies in ART program monitoring and evaluation processes would ensure more data was collected over a longer period of time, allowing more robust conclusions to be drawn.

Several other factors may affect the generalizability of these findings to persons eligible for ART nationally, and across sub-Saharan Africa. Admission to the program depended on standard national public-sector inclusion criteria and care was provided through a clinic at public-sector, primary-care site. However, since this was the first clinic to make ART available in the district, those referred are likely to have been sicker than the eligible population on average, overestimating the level of care required. As the national program is rolled-out the nature of the program, and thus the utilization pattern of patients, may change. Furthermore, this population had better access to healthcare than most persons living in high HIV-burden areas due to its proximity to a major city and tertiary health services.

## Conclusion

Despite these limitations, the evidence of this study suggests that starting patients on ART, even when their illness is advanced, can lead to a significant and rapid fall in healthcare demand, particularly for HIV-related illnesses. It further suggests that earlier commencement might avoid the cost of treating many of the OIs seen in this population.

## Competing interests

The author(s) declare that they have no competing interests.

## Authors' contributions

RW conceived the study and helped to draft the manuscript. GH designed the study, acquired the data, conducted the statistical analysis and drafted the manuscript. CO conducted the clinical analysis and helped to draft the manuscript. All authors read and approved the final manuscript.

## Pre-publication history

The pre-publication history for this paper can be accessed here:


